# Optical and Mechanical Properties of Self-Repairing Pectin Biopolymers

**DOI:** 10.3390/polym14071345

**Published:** 2022-03-26

**Authors:** Aidan F. Pierce, Betty S. Liu, Matthew Liao, Willi L. Wagner, Hassan A. Khalil, Zi Chen, Maximilian Ackermann, Steven J. Mentzer

**Affiliations:** 1 Laboratory of Adaptive and Regenerative Biology, Brigham & Women’s Hospital, Harvard Medical School, Boston, MA 02115, USA; afpierce@bwh.harvard.edu (A.F.P.); bliu15@bwh.harvard.edu (B.S.L.); mliao3@bwh.harvard.edu (M.L.); willi.wagner@med.uni-heidelberg.de (W.L.W.); hakhalil@bwh.harvard.edu (H.A.K.); zchen33@bwh.harvard.edu (Z.C.); 2 Translational Lung Research Center, Department of Diagnostic and Interventional Radiology, University of Heidelberg, 69117 Heidelberg, Germany; 3 Institute of Functional and Clinical Anatomy, University Medical Center of the Johannes Gutenberg-University Mainz, 55122 Mainz, Germany; maximilian.ackermann@uni-mainz.de

**Keywords:** pectin, cohesion, adhesion, self-repair, fractography

## Abstract

Pectin’s unique physicochemical properties have been linked to a variety of reparative and regenerative processes in nature. To investigate the effect of water on pectin repair, we used a 5 mm stainless-steel uniaxial load to fracture glass phase pectin films. The fractured gel phase films were placed on a 1.5–1.8 mm thick layer of water and incubated for 8 h at room temperature and ambient humidity. There was no immersion or agitation. The repaired pectin film was subsequently assessed for its optical and mechanical properties. Light microscopy demonstrated repair of the detectable fracture area and restoration of the films’ optical properties. The burst strength of the repaired film declined to 55% of the original film. However, its resilience was restored to 87% of the original film. Finally, a comparison of the initial and post-repair fracture patterns demonstrated no recurrent fissures in the repaired glass phase films. The water-induced repair of the pectin film was superior to the optical and mechanical properties of the repaired films composed of nanocellulose fibers, sodium hyaluronate, and oxidized cellulose. We conclude that the unique physicochemical properties of pectin facilitate the water-induced self-repair of fractured pectin films.

## 1. Introduction

Polysaccharide biopolymers including agarose [1], cellulose [2], alginate [3], chitin [4], and pectin [5] have been implicated in a variety of biomedical applications. Pectin is a particularly intriguing polysaccharide [6]. Pectin is a ubiquitous polysaccharide that contributes substantially to the planet’s biomass. Pectin also has unique chemical and functional features. Chemically, the most abundant component of pectin is homogalacturonan, a glycan of α1→4-linked D-galacturonic acid that can be carboxy methyl esterified [7,8]. Functionally, pectin is involved in the intercellular adhesion and mechanical resistance of the plant cell wall to a variety of environmental challenges [9]. More recently, pectin has been shown to bind the mesothelial glycocalyx of visceral organs [10], suggesting a potential role in mesothelial healing and repair [11,12,13].

Pectin’s ability to bind mesothelial surfaces reflects a more general property of pectin as a bioadhesive [10,11,14,15]. Although the mechanism of pectin adhesion is not well understood, the process appears to require a liquid (wetting phase) interface. The liquid interface facilitates contact and enhances the interaction between surfaces [16]. In mucoadhesion, the interaction between surfaces is further enhanced by the movement of water from the mucous layer to the pectin. This water movement has been associated with enhanced pectin adhesivity [17,18]. We and others have proposed that the adhesive phase involves the entanglement of the branched chains of the pectin polymer [19,20,21].

Pectin chain entanglement appears to have substantial clinical relevance. Recent work has demonstrated that the surface of visceral organs is covered with glycocalyx 100- to 1000-fold thicker than previously recognized [22]. The glycocalyx demonstrates a branched-chain structure that appears to be similar the structure of pectin [22]. The entangled pectin chains, reminiscent of commercial hook-and-loop fasteners [23], contributes to the strong adhesive bond to the organ surface [19]. The importance of water movement in entangling pectin chains suggests the relevance of a numerous array of membrane pits underlying the mesothelial glycocalyx [22]. In previous reports, we have speculated that these pits may provide a source of the fluid movement that facilitates the chain interaction between pectin and the native glycocalyx [19]. The sliding of pectin chains likely contributes to the remarkable flexibility and extensibility of pectin bound to the visceral organ surface [14,15,24].

In addition to potential application in biomedicine, pectin’s unique physicochemical properties have been linked to reparative and regenerative processes in plants. Secreted pectin contributes to the occlusion of xylem and the compartmentalization of decay in the process of tylosis [25,26,27]. Pectic substances appear to be secreted both within and across the tylosis primary wall [28]. The result is that pectin is a basic component of the protective plugging material in the vessel lumina of trees. Deposited pectin also supports the adhesion and cohesion between the adjacent cells in the early steps of plant grafting. Pectin polysaccharides are present at the cellular interface within the first week after grafting [29]. Pectins appear to be acting as a cementing material between adjacent cells [30]. The central role of pectin in cell-driven processes suggests that the intrinsic properties of the polymer may be relevant to the cell-free process of self-repair as well.

In this report, we investigated the role of water in repairing fractures of glass phase pectin films. The pectin films were evaluated for optical properties, cohesive strength, and resilience. Pectin was compared to biopolymers composed of nanocellulose fibers, sodium hyaluronate, and oxidized cellulose.

## 2. Methods

**Pectin**. The unmixed citrus pectins used in this study were obtained from a commercial source (Cargill, Minneapolis, MN, USA) and characterized by a glycosyl residue content of 76–87% galacturonic acid, 2–6% rhamnose, 8–15% galactose, and 0.8–5% arabinose based on gas chromatography–mass spectrometry of trimethylsilyl derivatives and a 78–86% homogalacturonan and 16–26% RGI content. The degree of methoxylation was determined by the proportion of galacturonic acid residues in the methyl ester form. We defined high methoxyl pectin (HMP) as those pectin polymers with a greater than 70% degree of methoxylation. The pectin powder was maintained in environmentally controlled low humidity storage at 25 °C.

**Pectin dissolution in water**. The pectin powder was dissolved by a graduated increase in added water to avoid undissolved powder [31]. The powder dissolution was performed at 25 °C. Swelling and softening of the particles was followed by gradual dissolution [32]. Complete dissolution of the pectin was achieved by a high-shear mixing with a 10,000-rpm rotor-stator mixer (L5M-A, Silverson, East Longmeadow, MA, USA). Plateau viscosity was monitored using a digital ammeter (DataLogger, Silverson, East Longmeadow, MA, USA). The dissolved pectin was poured into a variety of shaped polystyrene molds and cured for further studies [16].

**Nanocellulose fibers (NCF)**. Briefly, NCF was obtained from the Process Development Center at the University of Maine (Orono, ME, USA) [19]. The NCF dissolution was obtained with progressive hydration followed by high-shear 10,000 rpm rotor-stator mixer (L5M-A, Silverson). The NCF powder was dissolved at 25 °C by a gradual dissolution in a process similar to pectin. The dissolved NCF was poured into polystyrene molds and cured for further studies.

**Surgical films**. A comparison for pectin and NCF was provided by commercially available surgical films. SepraFilm (Genzyme, Cambridge, MA, USA), here referred to as hyaluronate, is a sodium hyaluronate and carboxymethylcellulose film designed as an adhesion barrier for use in abdominal surgery [33]. Surgicel (Surgicel, Ethicon, Neuchatel, Switzerland), here referred to as oxidized cellulose (ox-cellulose), is an oxidized regenerated cellulose patch commonly used in surgery for hemostasis [34].

**Glass and gel phase**. The empiric definition of glass phase was water content (*w*/*w*) of 5–15%. In contrast, the gel phase was defined as a water content (*w*/*w*) of 20–30%.

**Image analysis**. Image analysis was performed after initial processing with the MetaMorph Imaging System 7.9 software (Molecular Devices, Brandywine, PA). The 12-bit grayscale images were obtained by 12-megapixel CCD camera. The images were processed using standard MetaMorph filters. After routine calibration and thresholding, the optical defects in the pectin films were detected by the MetaMorph integrated morphometry application. The *Relative Total Area* and *Integrated Optical Density* functions were used to quantify film defects.

**Fracture mechanics**. Fracture mechanics were performed as previously described [16]. Briefly, the biopolymers were subjected to a controlled uniaxial load normal to the plane of the polymer film. This load was achieved with a 5 mm stainless-steel spherical probe mounted to a TA-XT plus (Stable Micro Systems) with a 50 kg load cell. The stainless-steel ball was positioned centrally over the biopolymer. The probe compressed the biopolymers at a test speed of 2 mm/s until fracture. The fracture force, distance, and time were recorded. The fractured films were imaged and analyzed as above.

**Resilience measurements**. Resilience testing was performed to determine the elastic energy absorbed by the pectin biopolymers (that is, the area under the elastic portion of the stress-strain curve). Resilience was measured using an TA-XT plus (Stable Micro Systems). After calibration of the 50 kg load cell, a 5 mm spherical probe descended at a test speed of 0.5 mm/s (5 gm trigger force) to a limit of 1 mm and was withdrawn at the identical speed. The ratio of the area under the force curve during compression (Area1) and withdrawal (Area2) was defined as resilience (Resilience = Area2/Area1).

**Scanning electron microscopy**. After coating with 20–25 A gold in an argon atmosphere, the pectin films were imaged using a Philips XL30 ESEM scanning electron microscope (Philips, Eindhoven, Netherlands) at 15 Kev and 21 μA. Stereo pair images were obtained using a tilt angle difference of 6˚ on a eucentric sample holder using standardized automation.

**Statistical analysis.** The statistical analysis was based on replicate measurements. Biologic and technical variance was systematically evaluated. The unpaired Student’s *t* test for samples of unequal variances was used to calculate statistical significance. Typically, the data were expressed as mean ± one standard deviation (SD). By convention, the significance level for a sample distribution was defined as *p* < 0.01.

## 3. Results

**Figure 1:**
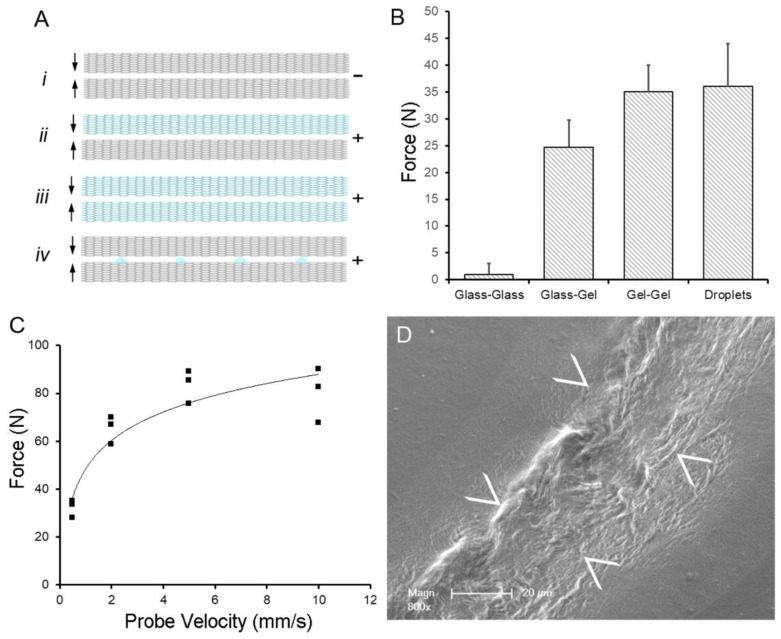
Water-induced adhesion of pectin films cured in parallel. (**A**) Schematic of pectin films in glass phase (gray) and gel phase (blue). The (–) indictes no adhesion, whereas (+) indicates an adhesive interaction between films. (**B**) Using a previously defined adhesion assay [35], pectin films were gently compressed (5N) for a 60 s development time followed by withdrawal of the films at 0.5 mm/s. Two glass phase films (A*i*) demonstrated no adhesion. Hydration of one (A*ii*) or both (A*iii*) of the films to 15% water content resulted in significant adhesion. An equal volume of water placed on the glass phase films as droplets (A*iv*) resulted in comparable adhesion to two gel films. (**C**) In the droplet assay, adhesion was largely independent of probe speed (that is, the speed at which the films were compressed). (**D**) Scanning electron microscopy (SEM) of two films following droplet compression shows evidence of polymer chain entanglement at the film interface (arrows at film interface).

**Water-induced blending of pectin films.** To investigate the effect of water on pectin polymer repair, we measured the adhesive strength between two high methoxyl pectin films cured in parallel and differing only in their water content. The films were gently compressed for 60 s and then separated. The measured withdrawal force was a reflection of polymer-polymer blending. Replicate studies demonstrated that there was no significant adhesion between two glass phase films (Figure 1A,B). In contrast, if one or both of the films were hydrated to gel phase, the adhesion was significant (*p* < 0.001). Interestingly, if a quantity of water equal to the difference between glass and gel phase films was introduced at the glass film interface, there was significant polymer-polymer adhesion (Figure 1B). The adhesion strength of the water droplet-blended films was largely independent of compression force (not shown) and probe compression velocity (Figure 1C). The effective blending the polymer films was confirmed by electron microscopy. SEM imaging of the interface between pectin films suggested the intermingling of pectin chains (Figure 1D).

**Figure 2:**
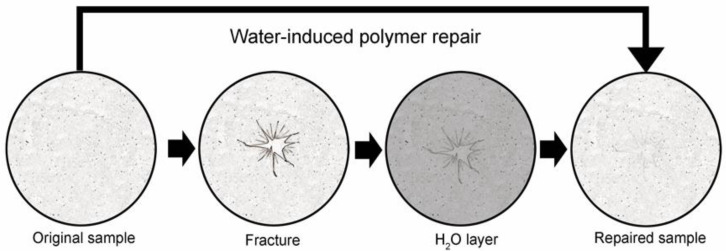
Schematic of water-induced repair of fractured pectin films. The glass phase pectin film is fractured using a controlled uniaxial load with a 5 mm stainless steel probe (2 mm/s). The fractured film is placed on a thin layer of distilled water (1.6 to 1.8 mm) for 8 h at 25 °C followed by optical and mechanical analysis of the repaired films.

**Figure 3:**
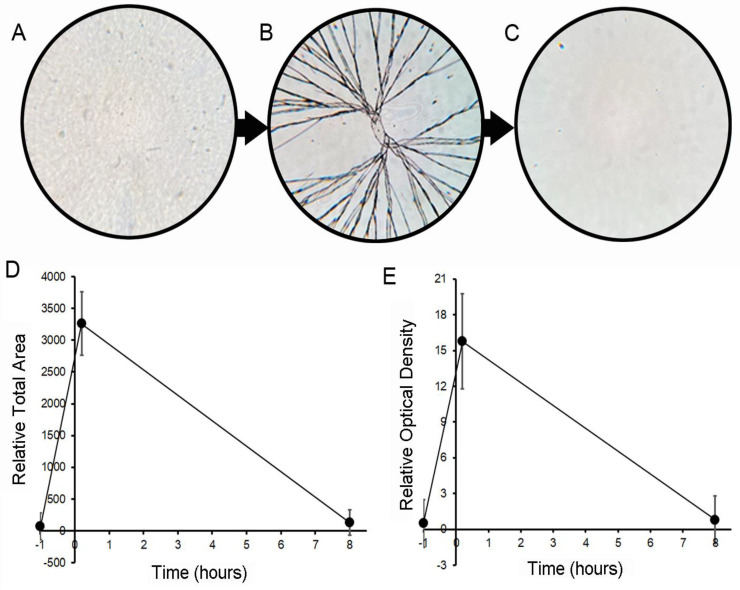
Optical properties of pectin films after fracture and water-induced repair. (**A**) A representative film was fractured (**B**) using a controlled uniaxial load with a 5 mm stainless steel probe (2 mm/s) and then underwent water repair (**C**). Epi-illumination light microscopy was used to image the films. After routine thresholding and processing with standard MetaMorph filters, the optical features were measured using MetaMorph’s integrated morphometry applications. Morphometric assessment of *Relative Total Area* (**D**) and *Relative Optical Density* (**E**) are dimensionless functions that indicated near-complete resolution of the fracture-associated optical defects after the eight-hour repair period. The mean ± 1 standard deviation (SD) of the replicate samples (N = 8) is shown. The optical properties were assessed at a water content of 8% (*w*/*w*).

**Repair of optical properties.** To investigate the potential for water-induced pectin self-repair, glass phase films were fractured with a controlled uniaxial load. A volume of water sufficient to cover the bottom of the mold was introduced (the water layer was 1.6 to 1.8 mm in depth). The water was introduced first to avoid inadvertent mixing. The film was incubated in nearly identical conditions to the initial curing for a minimum of 8 h at room temperature (25 °C) and in ambient humidity (Figure 2). There was no agitation or mixing. The resolution of the fractures was assessed by epi-illumination light microscopy and digital image analysis (Figure 3A–C). The repaired films demonstrated near-complete resolution of the fracture by optical measures of the total fracture area (Figure 3D) as well as the overall optical density (Figure 3E) of the film.

**Figure 4:**
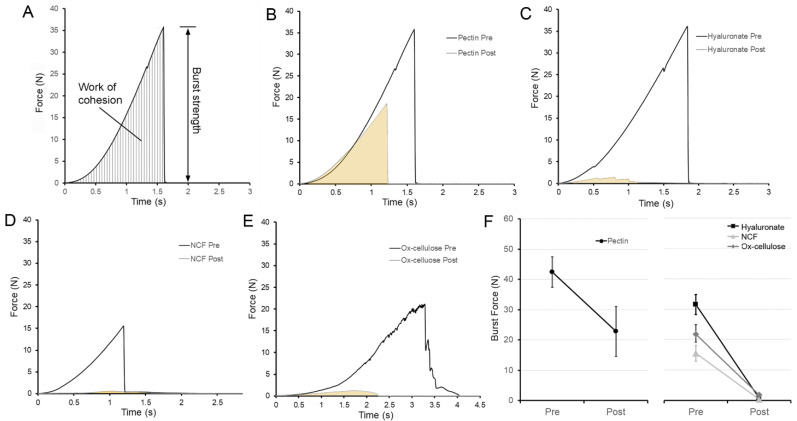
Burst strength and fracture mechanics of pectin films before and after water repair. (**A**) Using a spherical stainless-steel probe (5 mm), the pectin films were loaded (2 mm/s) until fracture. The peak force was recorded as burst strength. After fracture of the film, water-induced repair was followed by repeat fracture of the film. (**B**) Characteristic burst force curves of pectin films are shown before (black) and after (gray) burst fracture and water-induced repair. Burst strength curves are also shown for sodium hyaluronate (**C**), NCF (**D**), and ox-cellulose (**E**). (**F**) Summary of burst strength replicates (N = 10). Pectin burst strength after repair was significantly greater than the three controls (*p* < 0.001). The mean ± 1 standard deviation (SD) is shown. The fracture mechanics were assessed at a water content of 8% (*w*/*w*).

**Repair of the burst strength**. To test the mechanical strength of the repaired pectin film, a second fracture was created. The burst force required for the second fracture decreased to 55% of the original burst force (Figure 4B, *p* < 0.001). Despite the decrease in burst strength of pectin films, the repaired pectin films were significantly stronger than comparison biomedical polymers including NCF, hyaluronate, and ox-cellulose (*p* < 0.001) (Figure 4C–F).

**Figure 5:**
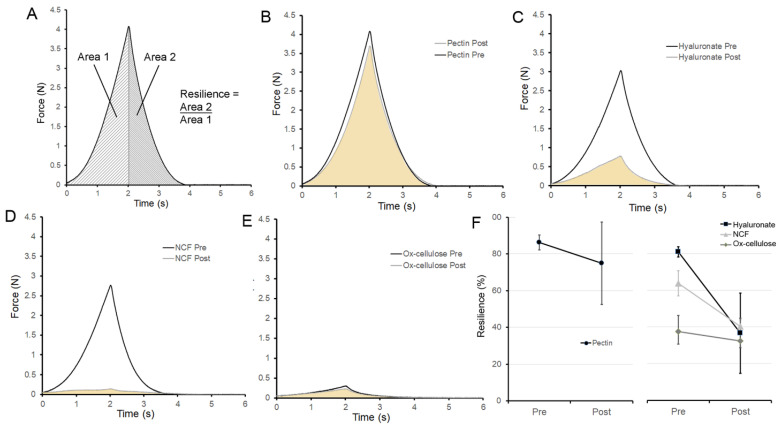
Resilience of pectin films before and after water-induced repair. (**A**) A 5 mm stainless steel sphere was used to probe the pectin films to a displacement of 1 mm. The resilience was calculated as the ratio between absorbed energy during elastic deformation (Area 1) and the released energy upon unloading (Area 2). (**B**) Characteristic resilience curve of pectin films is shown before (black) and after (gray) burst fracture and water-induced repair. Similar resilience curves are shown for sodium hyaluronate (**C**), NCF (**D**), and ox-cellulose (**E**). (**F**) Summary of resilience replicates (N = 10) is shown. Pectin resilience was significantly greater than the polymer controls (*p* < 0.001). The mean ± 1 standard deviation (SD) is shown. The resilience mechanics were assessed at a water content of 8% (*w*/*w*).

**Repair of resilience**. To test the ability of the repaired pectin films to withstand elastic deformation, the resilience of the repaired films was assessed. The stainless-steel probe deformed the films 1 mm recording both the deformation force and the stored energy released upon probe withdrawal. This ratio was defined as resilience (Figure 5A). Notably, the resilience of the pectin films was not significantly decreased after water-induced repair (Figure 5B, *p* > 0.01). The resilience of the repaired pectin film was 87% of the original film. Moreover, the restored pectin resilience was significantly greater than the other polymers tested (*p* < 0.001) (Figure 4C–F).

**Figure 6:**
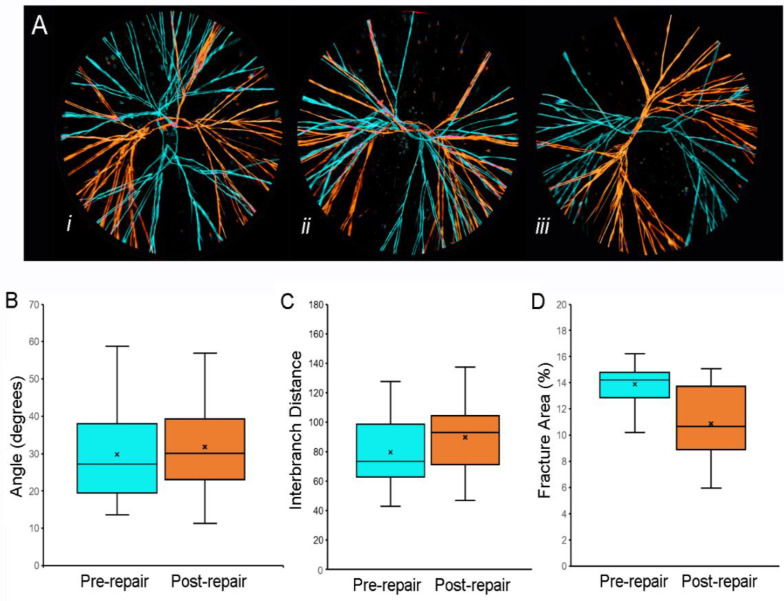
Fracture patterns of pectin films before and after water-induced repair. (**A**) Representative images of fracture patterns obtained before (blue) and after (orange) water-induced repair were thresholded and superimposed using the MetaMorph *Stack Arithmetic Logical XOR* function. Original orientation of the pectin films was maintained for comparison. The similar orientation of the fracture patterns in (A*ii*) was the exception; in most films, the fracture patterns appeared to be randomly oriented (e.g., (A*i*) and (A*iii*)). Morphometry of the fracture patterns demonstrated no significant difference in fracture angles (**B**), *p* > 0.05 or interbranch distance (**C**), *p* > 0.05. The total area of the pattern was slightly lower in the post-repair compared to the pre-repair fracture patterns (**C**), *p* < 0.05). In (**B**–**D**, the box spans the interquartile range with the median marked with an X and the whiskers defining the data range. A minimum of 10^3^ features were measured for each parameter.

**Repair fractography**. To identify areas of recurrent structural weakness, we compared the fracture pattern before and after water-induced repair. Representative overlays of fracture patterns obtained before (Figure 6A, blue) and after (Figure 6A, orange) water-induced repair are shown. Although the orientation of the second fracture pattern was occasionally similar to the original pattern (e.g., Figure 6(A*ii*)), the overlays demonstrated no recurrent fissures. Image analysis indicated that the fracture angles and interbranch distances were similar both pre- and post-repair (*p* > 0.05). Of unclear significance, the total area of the optically defined fracture area was marginally smaller after water-induced fracture repair (*p* = 0.05).

## 4. Discussion

In this report, the repair of fractured glass phase pectin films required exposure to a thin layer of water and an amount of time equal to the initial curing period (≥8 h). The addition of water and time was associated with several reparative changes. First, we observed repair of the visible fractures and restoration of the measurable optical properties of the film. Second, there was evidence of cohesive repair and a partial return of burst strength. Third, the repaired films demonstrated a near-complete restoration of the films’ initial resilience. Finally, the fracture pattern of the repaired film was distinct from the pre-repair fracture pattern. We conclude that the unique physicochemical properties of pectin facilitated the water-induced self-repair of fractured pectin films.

The water-induced repair of the pectin films is notable since the conditions were identical to initial curing (i.e., similar water content, time, and temperature). This modest intervention (the addition of a thin layer of water) resulted in the remarkable restoration of most of the films’ optical and mechanical properties. Although the mechanism is unclear, water-induced repair is likely dependent upon the chain-like structure of pectin polymers. We speculate that the water facilitated interdiffusion of these chains across the films’ fractures [36]. In contrast, comparable chain interdiffusion was not observed with the cellulose- and hyaluronate-based biopolymers.

Pectin, a fundamental structural element in plants [37], demonstrates a remarkable combination of both strength and toughness. In materials science, strength is generally measured as the energy required to cause fracture, whereas toughness is the material’s deformation resistance [38]. Here, water-induced repair nearly eliminated the optically detectable fissures in the translucent pectin film but did not completely restore the film’s original cohesive strength. The burst strength of the repaired film was approximately half (55%) of the strength measured after initial curing. This observation suggests that fracturing fundamentally perturbed the optimal polymer chain structure achieved with initial curing. Water alone could not restore this optimal structure―despite the comparable water content and repair time.

Although burst strength was reduced after fracture, resilience was notably restored after water-induced repair. Resilience is the ability of a material to absorb energy when it is elastically deformed and release that energy upon unloading. In our experiments, pectin resilience was approximately 87% of the value after original curing. There are many potential mechanisms to relieve locally high stresses and contribute to resilience, but we speculate that the sliding of pectin’s polymer chains facilitated by water-induced repair contributed to pectin’s intrinsic toughening and restored resilience.

In conclusion, we have shown that pectin films are capable of a remarkable degree of structural repair (a process triggered by exposure to a thin layer of water). This is a unique observation that suggests a physicochemical process of self-organization that deserves further study. In other biopolymer systems, the claim of self-healing is associated with the resolution of structural fissures and at least partially restoration of mechanical properties [39]. A crucial distinction is the cell-free conditions of our experiments. In most cases, self-healing is generally considered a cell-driven process [40]. Here, the reparative process was observed in isolated polymers studied in cell-free conditions. Since the repair process reflects the unique physicochemical properties of pectin, we suspect these observations justify the claim of water-induced self-repair.

## Data Availability

The data presented in this study are available on request from the corresponding author.

## Abbreviations

HMP: high methoxyl pectin; ox-cellulose: regenerated oxidized cellulose; pps: points per second; SD: standard deviation; SEM: scanning electron microscopy.
